# A Discussion on Hospital Construction

**Published:** 1887-11-19

**Authors:** 


### The Hopitals Association.

A Discussion on Hospital Construction.

On Wednesday evening, November 9th, the second meeting of the fifth session of the Hospitals Association was held, by kind permission of the Weekly Board, in the Board Room of the Middlesex Hospital. The chair was taken by Sir Douglas Galton, K.C.B. There was a crowded audience, many well-known architects and sanitarians in addition to medical practitioners and hospital managers being present.

The discussion on Mr. Young's paper on "Hospital Construction," published on p. 121, was opened by Sir Douglas Galton, K.C.B., F.R.S., who said: I am sure you will all be most ready to accord a vote of thanks to Mr. Keith Young for his interesting paper on "Hospital Construction." He has made a complete summary of most of those principles which have, no doubt, been long known and admitted by those who possess a knowledge of hospital construction. There is no subject, I think, at the present time which appears to have a greater interest for the community generally than this one of good sound principles in hospital construction. In England we have not, perhaps, pursued the matter to the same extent as it has been recently pursued on the Continent of Europe and in America; but I think that we have fully considered the arrangements which are best adapted to our climate and country in the recently constructed hospitals. There is no doubt that we have changed our opinions very much within the last twenty-five years, and I think that now the balance of opinion would be, if space permitted, in favour of having the various wards for the sick in isolated buildings of one storey high and connected by some form of protected corridor. But it is quite impossible to do that in a large town, and consequently we must resort to hospitals having a certain number of storeys one above the other. I agree with Mr. Young in his view that, if the wards are properly separated from each other by adequate floors, and provided their ventilation is properly carried out and their staircases not placed in such a position as to form shafts of communication between wards, you will be able to have perfectly healthy and safe wards placed one above another in a town. All the recent buildings of the Metropolitan Asylums Board are entirely upon the principle of separation of wards, but their buildings are for fever and small-pox patients, where isolation is, of course, of much greater importance than in other cases; but, however that may be, one point to be considered in your wards is how to give adequate floor space. That is a far more important point than any other, and it is one which should exercise the attention of the architect who designs hospitals. Now with regard to the form to be given to the ward, I will not enter this evening into the disputed question of circular *versus* rectangular wards. For wards of a limited number of beds, it is quite possible that the circular form may be a very satisfactory and a very agreeable one, but when you come to large wards of twenty-four beds or more then the difficulties of the circular system become very apparent. Of course the form of every ward is a question which must be decided with reference to the number of beds and perhaps with reference to certain other special arrangements. I would say upon the question of ventilation that I agree with Mr. Young that in this climate natural ventilation is quite a sufficient and satisfactory form of ventilation. Of course when you go to a place like New York, where the extremes of temperature are very great—intensely cold in the winter and hot in the summer—you are obliged to make special arrangements and ventilate the ward without having recourse to the opening of windows in the winter, and you equally desire to force cool air into the wards in summer. Their arrangements in New York for artificial ventilation are very ingenious and very complete, but they are also very expensive, and undoubtedly as long as our climate permits us to avoid that system of expensive ventilation we are prudent in not endeavouring to resort to it. There is one point in Mr. Young's paper where he speaks of separating the administrative entirely from the ward blocks. Now, I visited the other day the Tenon Hospital, in Paris. There the hospital undoubtedly covers a very large extent of ground, such as in a town like London it would be impossible to obtain, but the arrangements regarding the administration are very interesting and complete. There they distribute the whole of the provisions and the clean linen to the patients by means of subterranean corridors which communicate with the kitchen and other administrative buildings, which are separated from the ward buildings. Everything from the kitchen is taken by a lift, and is carried along under the various wards required. Thence it is taken up by hydraulic lifts to the different wards in the hospital, and by this means you never see anyone connected with the administration moving about between the wards of the hospital. I understand at Liverpool, in the new hospital, the architect has proposed to carry all the provisions to the different wards from the kitchen block above the hospital wards by means of raised corridors. He proposes to have the kitchen at the top of the administrative block, and to have lines of railways running along the tops of the corridors, and to drop the food down into the different pavilions. I myself should prefer the system of the Tenon Hospital at Paris, because you avoid having elevated corridors, which prevent the circulation of the air between the different pavilions. If you have the pavilion system, you should supplement it by having the corridors kept as low as possible, preventing any interference in the circulation of the air between different wards. I have been most interested in the paper which Mr. Keith Young has just read, and I certainly feel that a great debt of gratitude is due to him for having expressed so very well and tersely so many interesting points in Hospital Construction.

Mr. Michelli (Seamen's Hospital): With regard to what are called the Middlesex Hospital windows: At St. Mary's Hospital at Paddington they are made on this system, but they do not open all at once. The top, for example, can be opened, and the window can be opened down below. I remember there was a very interesting paper, which touched on a good deal of this subject, read by Mr. Theodore Williams at the Brompton Consumption Hospital last session, and then I opposed the idea of mechanical ventilation—ventilation through tubes. Mr. Young has not touched at all upon the accumulation of dust and dirt which gets into the tubes. I remember an instance in which a ventilator under a ward upon being opened was found to contain, among other things, a dead bird, so that the ventilator must have been carrying foul air into the ward for a long while.

Mr. Robins, F.S.A.: I think upon the points that Mr. Young has touched upon our views will be pretty well identical. Regarding ventilation, that is a most delightful subject upon which no two persons will be found to agree. With regard to external ventilation, I think the best mode of ventilation is that adopted at the Heidelberg Hospital. Heidelberg is very cold at certain times of the year, and thus I do not think we need be afraid to adopt the same thing here. I think you should have as few connected corridors as possible, and if you could do without them altogether, as at Heidelberg, so much the better. Regarding internal ventilation, I was much struck with a paper read some time since by Dr. Carnelli at Dundee. It was on the subject of the equality of the air in different schools in that town. Comparison was made for this reason—that a great number of schools had no system of ventilation at all, while others had an artificial system. The question was, which was the best? The result of the investigations which were made were strongly in favour of artificial ventilation. One thing I would like to say, and that is, whatever system of ventilation you adopt in bringing fresh air into hospital wards (if tubes cannot be kept clean), I can see no objection to their use for *extracting* the foul air. The air should come in through the walls directly and be warmed, as at the Brompton Consumption Hospital, where the air comes in through the window and passes over hot water pipes, and not through pipes at all.

Mr. Charles Bell, the architect of the Hampstead Infirmary, where there is one of the largest circular wards yet built, described the system of ventilation adopted there, which he believed acted perfectly, and an inspection of which he invited.

Mr. Gordon Smith (architect to the Local Government Board): I regard this subject as one of very great importance, because so much has recently been done in the matter, and so much yet remains to be done. During the last ten or fifteen years, in London alone there have been upwards of ten thousand beds provided for pauper sick patients only, leaving other hospitals out of the question, and also the accommodation for the infirm and aged poor. Very much the same rate of provision has been made all over the country. A good deal, however, yet remains to be done. Look at our hospitals in England and compare them with what we see abroad. I cannot help thinking that we are a long way behind the Continental hospital designers. In details, in sanitary arrangements, &c., we are no doubt in advance, but in the general distribution of our buildings we have a good deal yet to learn. Many of the Continental hospitals are only one storey high. The St. Denis Hospital, having between 160 and 170 beds, stands on six acres, while the site of the Heidelberg Hospital is perhaps ten, and that has only one storey. Liberality in the matter of site is the first element in hospital construction. In our English hospitals we crowd too much on a given area. The Chairman has referred to the system of connecting blocks together by subways. I object to subways under any conditions whatever; but if they must be provided, I would have them as at St. Denis. I am convinced that subways, such as those at St. Thomas's Hospital communicating with the cellars and distributing bad air, are arrangements which ought to be avoided as far as possible. What we know is possible in Berlin ought not to be impossible in England, for the climate there is not different from ours. The state of the weather in Berlin is published in the *Times* every day, and I have watched it for a long time, and find that it corresponds pretty much with our own. The Chorlton Union Hospital, in Manchester, built twenty years ago, was the first workhouse hospital erected on the pavilion principle with accommodation for 700 patients. There, there are seven or eight pavilions all connected by a brick covered-in way, with a terrace on the top which can be used by the patients, and I cannot hear of any complaints. The administrative arrangements needed some attention, but there was nothing which could not be got over. As regards the extent of land available, it is sometimes said that land in France and Germany is cheaper than here, but that is not altogether borne out by the facts. St. Denis cost £1,271 per acre; the hospital at Halle £1,125 per acre, so that it is not, you see, altogether a question of cheap land. There is an arrangement at the hospital at Halle which, to some extent, meets the difficulty about the covered-in way. That institution is divided not only into pavilions, but, so to speak, into small hospitals for surgical cases, or medical cases, for eye and ear cases, for diseases of women, and other cases. The principal sub-divisions are the surgical and the medical cases, and here the division comprises an administrative block with corridors connecting two pavilions, so you have, as it were, small hospitals combined. This arrangement meets half-way objectors to the complete absence of corridors. There is a good deal to be said regarding the details of these hospitals. As regards ventilation, I have noticed what I think is advantageous in some foreign hospitals. They all have far greater power of heating than is customary in English hospitals, and by these means they are able to have a greater amount of ventilation than we have in some of our hospitals. On the other hand, their ventilating arrangements are in some cases exceedingly complex, and compare unfavourably with ours.

(*To be continued*).

